# Application of Objective Structured Teaching Examination (OSTE) in Assessing Classroom Teaching Skills for Nursing undergraduates: A Quasi-experimental Study

**DOI:** 10.17533/udea.iee.v42n2e16

**Published:** 2024-07-10

**Authors:** Duan Pei, Hou Ping, Liu Lin, Shuang Qiu

**Affiliations:** 1 Nurse Educator, Masters. Lecturer. Email: 007609@yzu.edu.cn Yangzhou University China 007609@yzu.edu.cn; 2 Nurse Educator, Masters. Lecturer. Email: pinghou@yzu.edu.cn Yangzhou University China pinghou@yzu.edu.cn; 3 Nurse Educator, Ph.D. Professor. Email: liulin163com@163.com Yangzhou University China liulin163com@163.com; 4 Doctor, Masters. Professor. Email: qsmk361@sina.com. Corresponding author. Yangzhou hospital of traditional Chinese medicine China qsmk361@sina.com; 5 Nursing School of Yangzhou University, Yangzhou, China. Yangzhou University Nursing School Yangzhou University Yangzhou China; 6 Yangzhou hospital of traditional Chinese medicine, Yangzhou, China. Yangzhou hospital of traditional Chinese medicine Yangzhou hospital of traditional Chinese medicine Yangzhou China

**Keywords:** teaching, students, nursing, valuation of research programs and tools, enseñanza, estudiantes de enfermería, evaluación de programas e instrumentos de investigación, ensino, estudantes de enfermagem, avaliação de programas e instrumentos de pesquisa

## Abstract

**Objective.:**

To evaluate the pedagogical skills of third-year nursing students at Yangzhou University (China).

**Methods.:**

A multisite quasi-experimental design was used in this study. Fifty-five participants were selected by convenience sampling. The Objective Structured Teaching Evaluation (OSTE) scale was used to assess teaching skills. The evaluation included four different stages: Teaching Background Analysis (E1), Lesson Plan Presentation (E2), Mock Class (E3) and Teaching Reflection (E4). Prior to the assessment, the teachers assigned homework to the students to complete at the four stations.

**Results.:**

Fifty-five nursing students with an average age of 21.3±0.7 years participated in the study, with a predominance of female students (78.2%). The highest mean score was achieved in E1 (83.1), followed by E2 and E3 (82.5 and 82.3 respectively), while the lowest mean score was found in E4 (79.6). In E3, instructors gave lower scores for class organisation, class characteristics and overall performance compared to the self-reported scores of the standardised students (p<0.05). More than 80% of the students strongly agreed and recommended the OSTE as the primary method for assessing teaching skills in the classroom.

**Conclusion.:**

Deficits in teaching skills were identified in the participating students; this information will allow specific interventions to improve the situation. The OSTE instrument was a useful method for assessing the pedagogical skills of undergraduate nursing students.

## Resumen

**Objetivo.:**

Evaluar las habilidades pedagógicas de los estudiantes de tercer curso de enfermería de la Universidad de Yangzhou (China).

**Métodos.:**

En este estudio se empleó un diseño cuasi-experimental multisitio. Por muestreo por conveniencia se seleccionaron 55 participantes. Se empleó la escala Examen Objetivo Estructurado de la enseñanza (OSTE- Objective Structured Teaching Evaluation, en inglés) para evaluar las habilidades pedagógicas. La evaluación abarcó cuatro estaciones distintas: Análisis de los antecedentes de la enseñanza (E1), Presentación del plan de la lección (E2), Clase simulada (E3) y Reflexión sobre la enseñanza (E4). Antes de la evaluación, los instructores asignaron tareas a los estudiantes que debían realizar en las cuatro estaciones.

**Resultados.:**

Participaron en la investigación 55 estudiantes de enfermería con un promedio de edad fue de 21.3±0.7 años y predominó el sexo femenino (78.2%). La puntuación media más alta se alcanzó en la E1 (83.1), seguida por E2 y E3 (82.5 y 82.3, respectivamente, mientras que la puntuación media más baja se registró en la E4 (79.6). En la E3 los instructores asignaron puntuaciones más bajas en comparación a las autorreportadas por los estudiantes estandarizados en términos de organización de la enseñanza, características de la enseñanza y rendimiento general (*p*<0.05). Más del 80% de los estudiantes se mostraron totalmente de acuerdo y recomendaron el OSTE como método principal para evaluar las habilidades docentes en el aula.

**Conclusión.:**

Se identificaron en los estudiantes participantes las deficiencias en las habilidades pedagógicas; esta información permitirá realizar intervenciones específicas para mejorar situaciones específicas de la situación encontrada. El instrumento OSTE fue un método útil para la valoración de las habilidades pedagógicas de los estudiantes universitarios de enfermería.

## Resumo

**Objetivo.:**

Avaliar as competências pedagógicas dos estudantes do terceiro ano de enfermagem da Universidade de Yangzhou (China).

**Métodos.:**

Um desenho quase-experimental multisite foi utilizado neste estudo. Por amostragem de conveniência, foram selecionados 55 participantes. A escala *Objective Structured Teaching Evaluation* (OSTE) foi utilizada para avaliar as competências pedagógicas. A avaliação abrangeu quatro estações distintas: Análise do percurso docente (E1), Apresentação do plano de aula (E2), Aula simulada (E3) e Reflexão sobre o ensino (E4). Antes da avaliação, os instrutores atribuíram tarefas aos alunos, que deveriam completar as quatro estações consecutivamente.

**Resultados.:**

Participaram da pesquisa 55 estudantes de enfermagem com idade média de 21.3±0.7 anos e predominou o sexo feminino (78.2%). A maior pontuação média foi alcançada em E1 (83.1), seguida de E2 e E3 (82.5 e 82.3, respectivamente, enquanto a menor pontuação média foi registrada em E4 (79.6). Em E3 os instrutores atribuíram pontuações mais autorrelatado por alunos padronizados em termos de organização do ensino, características de ensino e desempenho geral (*p*<0.05). Mais de 80% dos alunos concordaram fortemente e recomendaram o OSTE como principal método para avaliar habilidades de ensino em sala de aula.

**Conclusão.:**

Foram identificadas deficiências nas competências pedagógicas dos alunos; esta informação permitirá realizar intervenções específicas para melhorar situações específicas da situação encontrada. O instrumento OSTE foi um método útil para avaliar as competências pedagógicas de estudantes universitários de enfermagem.

## Introduction

Conventional assessment techniques are inadequate in accurately gauging students' teaching skills. As the demand for healthcare continues to increase, the role of nurses is constantly evolving to encompass not only providing care, organization, and management, but also educational responsibilities.^1^^,^^2^ In China, the development of preliminary teaching competence has become a key objective in the education of undergraduate nursing students. Many nursing schools in the country offer courses in nursing education and have implemented teaching reforms, such as the use of flipped classrooms.^3^^,^^4^ However, the commonly utilized assessment methods, both domestically and internationally, have relied on closed-book exams, which primarily focus on theoretical knowledge and do not directly assess students' teaching abilities. Consequently, there is considerable debate and a pressing need to establish a structured and standardized approach for evaluating students' teaching skills.

OSTE is commonly utilized for assessing clinical teaching competencies. OSTE, an acronym for Objective Structured Teaching Evaluation, encompasses a range of methodologies centered on standardized students, standardized clinical teaching scenarios, multisite assessment, and objective evaluation with the goal of enhancing the instructional capabilities of clinical educators.^5^ The concept originated from the Objective Structured Clinical Examination (OSCE), with Simpson *et al.*^6^adapting a similar approach to OSTE by employing Standardized Students (SS) to simulate standardized ambulatory teaching situations (SATS) to aid educators in improving their observation and feedback skills. Alternative interpretations for the "E" in OSTE, such as "encounter"^7^ or "examination",^8^ have been proposed by scholars. However, OSTE has emerged as the primary method for evaluating clinical teaching competence. Wilkes *et al*. ^9^ developed a three-year training program aimed at enhancing community faculty teaching skills, doctor-patient communication abilities, and clinical handling skills, with over 70% of the community faculty expressing belief in the efficacy of this assessment method for enhancing teaching abilities. 

A systematic review by Trowbridge in 2011, covering 22 studies on OSTE from 1975 to 2010, concluded that there was moderate evidence to support the use of OSTE as a method for assessing the teaching abilities of clinical educators.^10^ Subsequently, OSTE has been adapted and applied in various contexts within health professional education.^11^^-^^13^ Mahoney *et al*.^5^ utilized OSTE for the training and assessment of resident teaching skills, with results indicating strong acceptance of OSTE by participants and a demonstration of robust teaching self-efficacy. This suggests that OSTE is a scientifically valid and feasible tool for evaluating students' teaching skills.

The OSCE is anticipated to serve as a valuable method for evaluating students' proficiency in teaching. OSTE does not represent a distinct assessment approach; rather, it presents an objective, structured, and organized assessment framework. Within this framework, each assessment unit has the capacity to integrate suitable assessment content and methods in accordance with its teaching and examination guidelines. OSTE provides objectivity, standardization, and adaptability in the evaluation of teaching skills. Consequently, this investigation explores the utilization of OSTE in appraising teaching competencies within the context of the Nursing Education course, with the objective of comprehending students' teaching capabilities and investigating the potential implementation of this approach.

## Methods

Research Design. (i) *Assembling a research team*. The research team is composed of three instructors from the Nursing Education course, three nursing professors, and three students. Their primary responsibility is to supervise the quality and advancement of the study; (ii) *Crafting the research plan*. The research team collaborates to develop an OSTE implementation plan, with a primary focus on identifying research sites and organizing task assignments for each stage of assessment. OSTE is not a specific assessment method, but rather a structured and organized assessment framework. Within this framework, appropriate assessment content and methods can be included based on teaching and exam guidelines. Boendermaker *et al.*^14^ conducted interviews with general education faculty and identified six core skills related to teaching abilities: effective feedback skills, willingness to provide feedback, critical thinking about the learning process, good communication, respect for students, and stimulating students' thinking. To more accurately evaluate students' abilities in these areas, China's national conditions and teacher experiences were integrated. Following two expert meetings, four stations were designed, and scoring tools were developed for each station (refer to Table 1 for further details); (iii) *Establishing an assessment environment*. This study arranges three rooms and four assessment stations. Standardized students are recruited from across the university to participate in the study and are provided with training before the assessment 

**Table 1:** Station descriptions

Station	Case name	Station descriptions	Assessment Competence	Standardised student
1	Teaching Background Analysis	The assessors present an overview of the students' learning situation using slide presentations	Cognitive Ability Analytical Ability	No
2	Lesson Plan Presentation	The assessors submit their lesson plans to the main examiner on the day of the assessment	Analytical Ability Design Ability	No
3	Simulated Classroom	The assessors, acting as students-turned-teachers, deliver the lesson and handle any unexpected teaching incidents.	Communication Ability Organizational Ability Interpersonal Ability	Yes
4	Teaching Reflection	The assessors provide a teaching reflection report and answer questions from the examiners after the simulated classroom session	Cognitive Ability Analytical Ability	No

Note: The assessment content, format, examination stations, and scoring criteria will be announced two weeks prior to the assessment.

Study Participants. In April 2023, this study utilized a cluster sampling method to select a cohort of third-year undergraduate nursing students from Yangzhou University in Yangzhou, China. 

OSTE Assessment Content and Methods. (i) *Site Design*. The evaluation comprises four components. *The first station*, *Teaching Background Analysis*, involves assessors presenting an overview of the students' learning situation through slide presentations. *At the second station, assessors submit their lesson plans* to the main examiner on the day of the assessment in a Lesson Plan Presentation. *The third station features a Simulated Classroom*, where standardized students (SS) are recruited to create an authentic teaching scenario. Assessors, acting as students turned teachers, deliver the lesson and manage any unexpected teaching incidents. *The fourth station*, *Teaching Reflection*, requires assessors to provide a teaching reflection report and respond to questions from the examiners following the simulated classroom session. (For further details, please refer to Table 1); (ii) *The scoring tables* at each station are developed by the research team. The assessment criteria for the four stations are as follows: The first station's assessment table for student analysis primarily includes five dimensions: learning ability (20 points), starting point of learning (20 points), learning status (20 points), class situation analysis (20 points), and textbook analysis (20 points). The second station's assessment table for lesson presentation mainly encompasses six dimensions: objective design (20 points), content analysis (10 points), instructional process design (40 points), extension design (10 points), document standardization (10 points), and innovative design (10 points). The third station's assessment table for simulated classroom consists mainly of five dimensions: teaching content (20 points), pedagogical organization (40 points), linguistic pedagogy (20 points), teaching characteristics (10 points), and courseware development (10 points). The fourth station's assessment table for teaching reflection covers four dimensions: teaching objectives (25 points), teaching philosophy (25 points), teaching methods (25 points), and teaching process (25 points); (iii) *Examiners*. Two assessors are designated to each station for this evaluation. In the third phase, standardized students (SS) participate in the assessment process. A total of 57 students were enlisted from the school for this evaluation.

Evaluation Process. Two weeks prior to the assessment, a briefing session is held with students to familiarize them with the OSTE process and furnish them with relevant instructions. The assessment content, format, examination stations, and scoring criteria will be announced at this time. On the day of evaluation, students undertake the assessment at each station under the supervision of examination personnel. With the exception of the third station, which spans 15 minutes, all other stations are brief, lasting only 3 minutes.

Quality Control. The research team is in charge of creating the implementation strategy for this OSTE. Both examiners and senior staff are knowledgeable about the assessment process, scoring criteria, and their specific duties. Examiners are tasked with scoring the first, second, and fourth stations, while the third station is assessed by both examiners and all senior staff, and the average score is used as the final score for each station. 

Ethical Issues. The study has obtained informed consent from the participants. This study has been approved by the Ethics Committee of the Nursing College of Yangzhou University (Ethical number: YZUHL20220122).

Questionnaire Survey. The research team develop

ped a survey to collect feedback from students who participated in the assessment. The survey includes four areas to assess the overall situation of OSTE: "The innovative assessment format," "Adaptability to this type of assessment," "Enhancement of teaching abilities during the assessment," and "Effective demonstration of teaching abilities through this format." The rating scale has five levels: "Strongly Agree, Agree, Neutral, Disagree, Strongly Disagree." Additionally, the survey asks if participants would recommend using OSTE as the primary method for assessing teaching abilities in nursing education courses, with options including "Definitely Not Recommend," "Not Recommend," "Neutral," "Recommend," and "Strongly Recommend." The survey is administered after students have completed all assessment stations.

Statistical Methods. We used SPSS Statistics 28 (IBM Corp, Armonk, NY) to perform statistical analyses. The independent samples t-test was used to compare means between groups, resulting in a significant *p*-value of 0.05.

**Figure:**
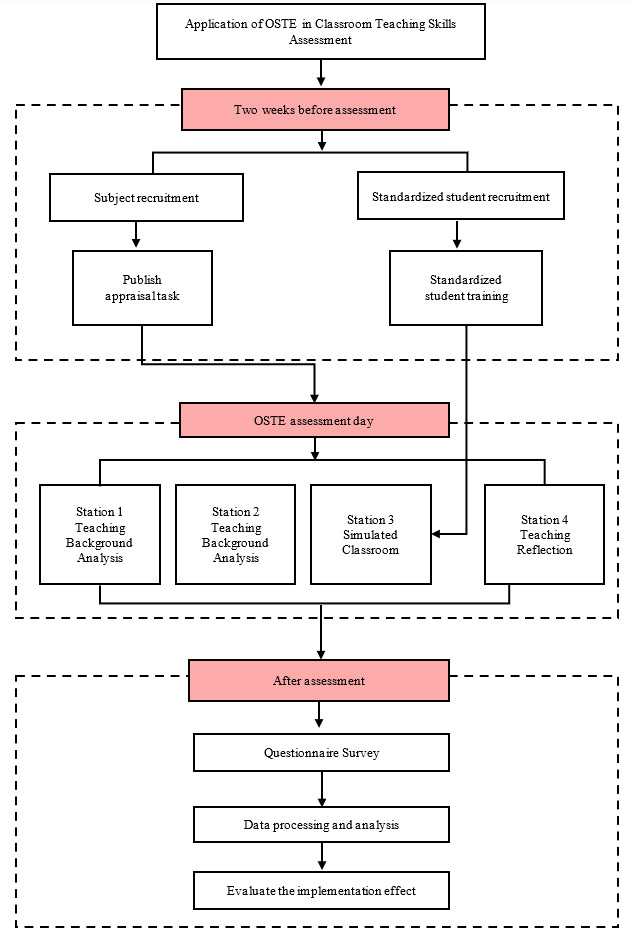

## Results

General Information. A study involved 55 undergraduate nursing students, with an average age of 21.3±0.7 years, of these, 43 (78.2%) were female. Additionally, 57 SS students from different fields such as education and horticulture participated, with an average age of 20.67 ± 1.03 years. Among them, 33 (57.89%) were female and 24 (42.11%) were male. 

OSTE Scores and Comparisons. The Student Teaching Background Analysis station had the highest score of 83.1±10.6, while the Teaching Reflection station had the lowest score of 79.7±10.3. In the simulated classroom stations, teachers received lower scores than standardized students in teaching organization, teaching characteristics, and overall performance. Conversely, standardized students scored lower than teachers in language and teaching demeanor, and these differences were statistically significant (*p*＜0.05). (Table 2).

**Table 2:** OSTE Scores and Comparison

Station	Dimension 1	Dimension 2	Dimension 3	Dimension 4	Dimension 5	Dimension 6	Total Score
Station 1	*Learning ability*	*Starting Point of Learning*	*Learning status*	*Class situation analysis*	*Textbook Analysis*	-	83.1±10.6
Mean ± SD	16.1±3.4	16.4±3.1	16.8±2.6	16.4±3.4	17.3±2.5	-	
MD	15	16.3	17.5	16.3	16.2		87.5
Station 2	*Objective Design*	*Content Analysis*	*Instructional Process Design*	*Extension Design*	*Document Standards*	*Design Innovation*	82.5±9.9
Mean ± SD	15.9±3.3	8.7±1.5	32.5±4.8	8.3±1.3	8. 7±1.2	8.4±1.3	
MD	15	9.37	35	7.5	8.8	7.5	82.6
Station 3	*Teaching Content*	*Pedagogical Organization*	*Linguistic Pedagogy*	*Teaching Characteristics*	*Courseware Development*	-	
Mean ± SD	17.0±3.1	31.3±4.7	17.5±2.4	8.1±1.1	8.4±1.3	-	82.3±9.1
*#*MD	16.3	30	17.5	8.12	7.5		83.1
Mean ± SD	*16.6±3.7	32.8±7.9	16.6±3.9	8.2±2.0	8.4±1.9	-	82.6±17.0
MD	15	30	15	7.5	7.5		85
t-test	t=0.424 *p*=0.331	t=0.796 *p* =0.002	t=0.920 *p*=0.002	t=0.078 *p*=0.009	t=0.158 *p*=0.125		t=0.060 *p*=0.002
Station 4	*Teaching Objectives*	*Pedagogical Philosophy*	*Teaching Methods*	*Teaching Process*	-	-	
Mean ± SD	19.5±3.3	18.0±2.7	21.5±3.6	20.7±4.3	-	-	79.7±10.3
MD	18.8	18.8	21.9	20.3			81.3

Note:"#" This line represents the standardized student’s rating; "-" indicates that the dimension is not applicable to this site, and "*" in one line represents standardizing student scoring in Station 3.

Assessment Score Sheet Following Evaluation. The assessment format received strong approval from over 80% of students, who highly recommend it as the main method for evaluating classroom teaching abilities.

**Table 3:** Assessment Score Sheet After Evaluation (*n*=55)

Indicator	Strongly Agree *n* (%)	Agree *n* (%)	Neutral *n* (%)	Disagree *n* (%)	Strongly Disagree *n* (%)
The assessment format is innovative	46 (83.6)	6 (10.9)	3 (5.5)	0	0
Being able to adapt to this type of assessment	45 (81.8)	10 (18.2)	0	0	0
Being able to adapt to this type of assessment," "Improvement of teaching abilities while undergoing the assessment	47 (85.5)	8 (14.5)	0	0	0
Effective demonstration of teaching abilities through this format	46 (83.6)	9 (16.4)	0	0	0
whether the participants would "recommend using OSTE as the primary method for assessing teaching abilities in nursing education courses."	44 (80)	8 (14.5)	3 (5.5)	0	0

## Discussion

OSTE demonstrates a high level of acceptance in evaluating the teaching abilities of students. In traditional terms, OSTE is a framework utilized to assess the teaching capabilities of clinical educators in the training of clinical educators or residents (15-17). However, its use has expanded beyond the medical field to include evaluating doctoral supervisors in the basic sciences, dental and pharmacy faculty, faculty portfolio coaches for medical students, medical student peer tutors, and Health Professions Education program graduates.^13^^,^^18^^-^^20^ OSTE has become a widely used method for assessing teaching skills in health professional education.^21^^,^^22^ Our study aimed to examine the effectiveness of using an enhanced OSTE to evaluate classroom teaching skills in nursing education. Four stations were created to evaluate students' cognitive, analytical, design, communication, organizational, and interpersonal skills, and overall, more than 80% of the students found the assessment form acceptable and recommended it as the primary method for evaluating classroom teaching skills. 

tilizing the OSTE offers a relatively comprehensive means of assessing students' pedagogical abilities. Previous research has placed less emphasis on assessing students' teaching skills, but the OSTE has been proven to significantly improve the clinical skill perception and confidence of test takers,^23^ enhance the skills of attending physicians 17, and improve the clinical teaching ability of educators.^24^ Bajwa *et al*.^25^ created a four-station OSTE assessment to evaluate participants' clinical teaching skills, with results showing that those in the coaching group displayed superior performance in various teaching aspects. Our findings reveal that students performed best in the Teaching Background Analysis station, while their lowest scores were in the Teaching Reflection station, indicating stronger cognitive and analytical skills compared to immediate feedback abilities. The simulation teaching station was rated as average, with teachers scoring lower than standardized students in teaching organization, characteristics, and overall scores. Standardized students scored lower than teachers in language style grading, showing that teachers were more stringent in evaluating teaching organization and characteristics, while students focused more on language and style of instruction delivery. Overall, the OSTE is a widely accepted and comprehensive method for evaluating students' classroom teaching ability, offering a promising and objective approach for assessing nursing students' professional skills. This study demonstrates the feasibility and acceptance of OSTE.

Strengths and Limitations. Our research has two key strengths. Firstly, we utilized the OSTE examination format, known for its objectivity and comprehensiveness. Additionally, we incorporated teaching ability components into OSTE to create a new assessment aligned with classroom teaching content. The assessment of teaching ability is thorough. However, the study's sample size is relatively small and derived from a single school dataset, which may limit its representativeness. Additionally, small sample studies often have lower statistical power, making it challenging to detect genuine differences. Furthermore, while OSTE can capture data at a specific point, it cannot consider longitudinal data within the nursing pedagogy process, limiting its ability to provide a comprehensive understanding of changes in teaching skills among nursing graduates.

Conclusions. The current research incorporates the evaluation of teaching skills in nursing education, with over 80% of students expressing support for this assessment method. This indicates that implementing OSTE enables students to recognize teaching skill weaknesses, leading to targeted interventions and improvements. However, the study has limitations. Firstly, the small sample size of students, examiners, and simulation scenarios made it challenging to detect statistically significant differences. Additionally, the limited number of OSTE sites may impact the assessment of students' teaching abilities. To obtain more reliable results, it is essential to refine the OSTE assessment content, increase the sample size, and expand the number of OSTE sites in future research. The study also proposes the development of OSTE-based training programs and the integration of OSTE coaching methods into instructional practices to enhance students' teaching abilities more effectively.
